# Supplementary figures and images for: Beta human papillomavirus 8E6 promotes alternative end joining
Source: eLife. 2023 Jan 24;12:e81923. doi: 10.7554/eLife.81923 (PMC9897725; doi:10.7554/eLife.81923)

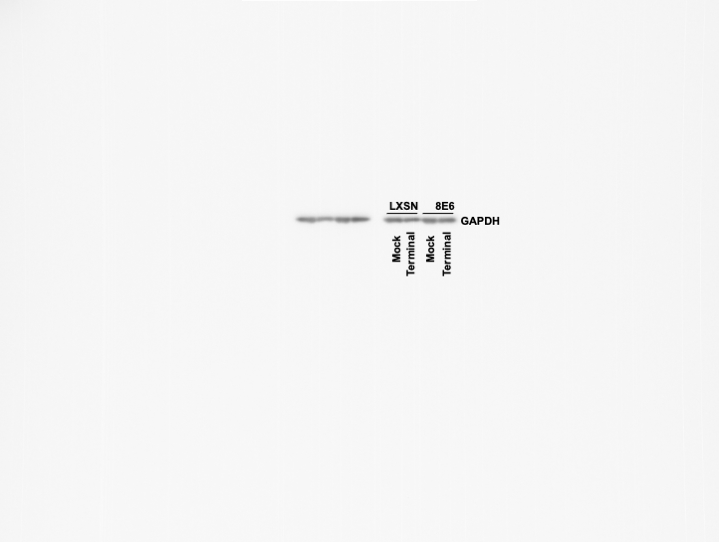

Supplement: Figure 1—figure supplement 1—source data 1. [file elife-81923-fig1-figsupp1-data1.zip › Figure 1-figure supplement 1 -source data/Figure 1-figure supplement 1A GAPDH.tiff]

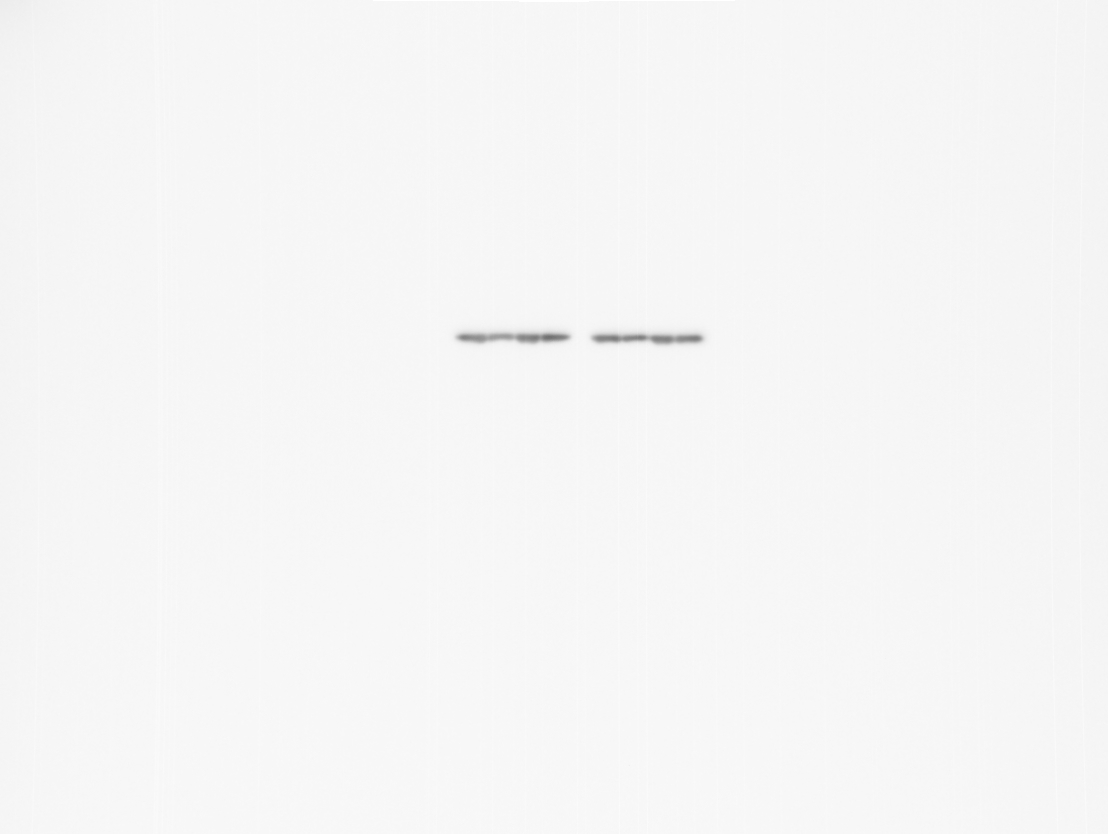

Supplement: Figure 1—figure supplement 1—source data 1. [file elife-81923-fig1-figsupp1-data1.zip › Figure 1-figure supplement 1 -source data/Figure 1-figure supplement 1A GAPDH.jpg]

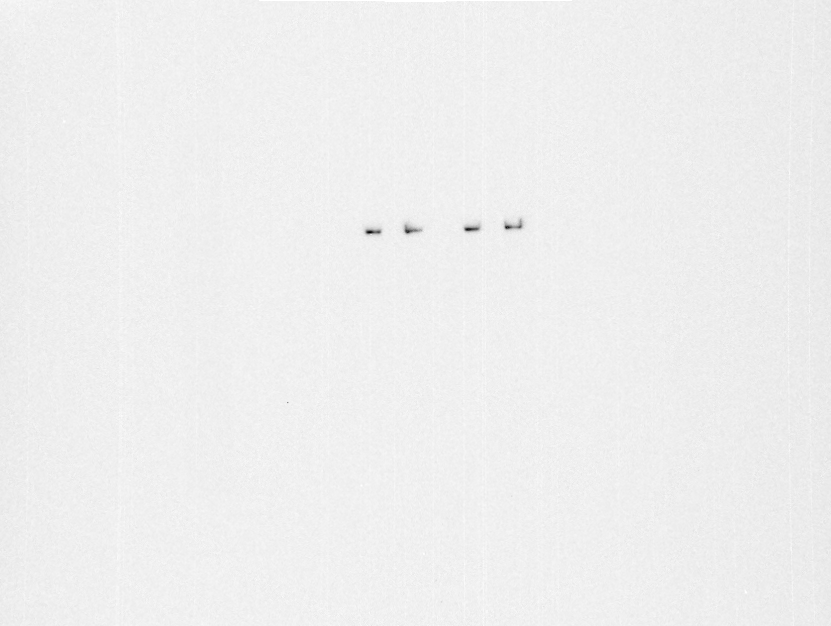

Supplement: Figure 1—figure supplement 1—source data 1. [file elife-81923-fig1-figsupp1-data1.zip › Figure 1-figure supplement 1 -source data/Figure 1-figure supplement 1C CAS9.jpg]

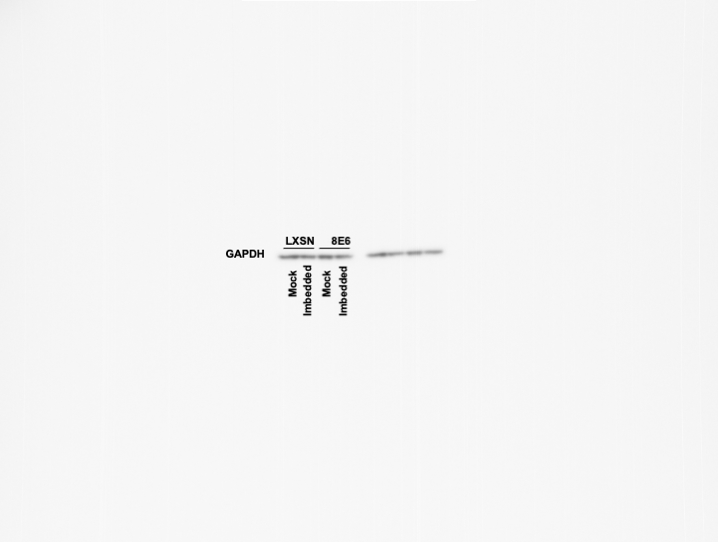

Supplement: Figure 1—figure supplement 1—source data 1. [file elife-81923-fig1-figsupp1-data1.zip › Figure 1-figure supplement 1 -source data/Figure 1-figure supplement 1C GAPDH.tiff]

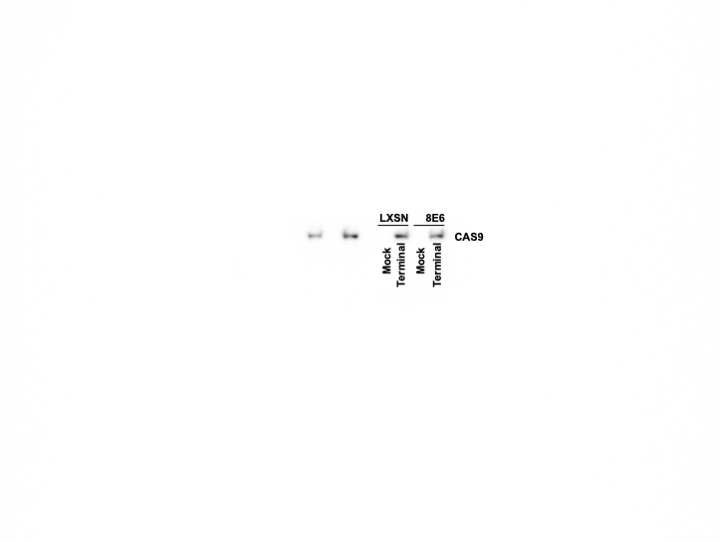

Supplement: Figure 1—figure supplement 1—source data 1. [file elife-81923-fig1-figsupp1-data1.zip › Figure 1-figure supplement 1 -source data/Figure 1-figure supplement 1A CAS9.tiff]

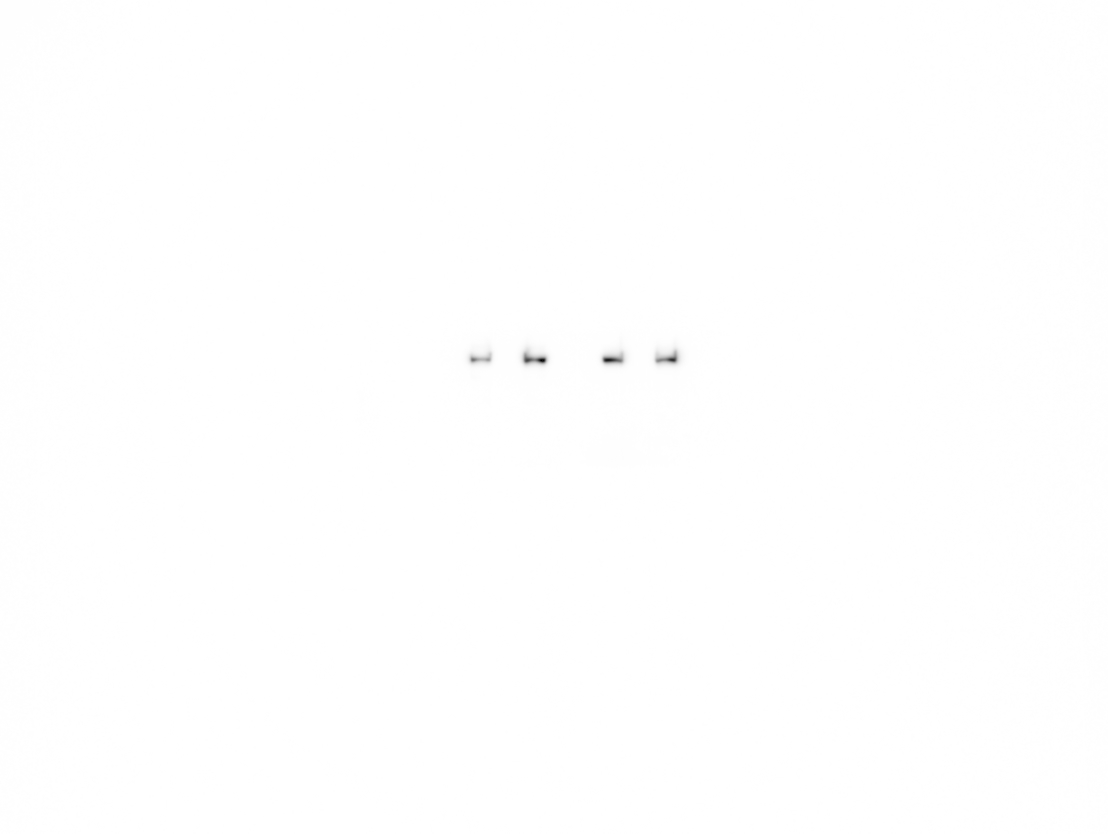

Supplement: Figure 1—figure supplement 1—source data 1. [file elife-81923-fig1-figsupp1-data1.zip › Figure 1-figure supplement 1 -source data/Figure 1-figure supplement 1A CAS9.jpg]

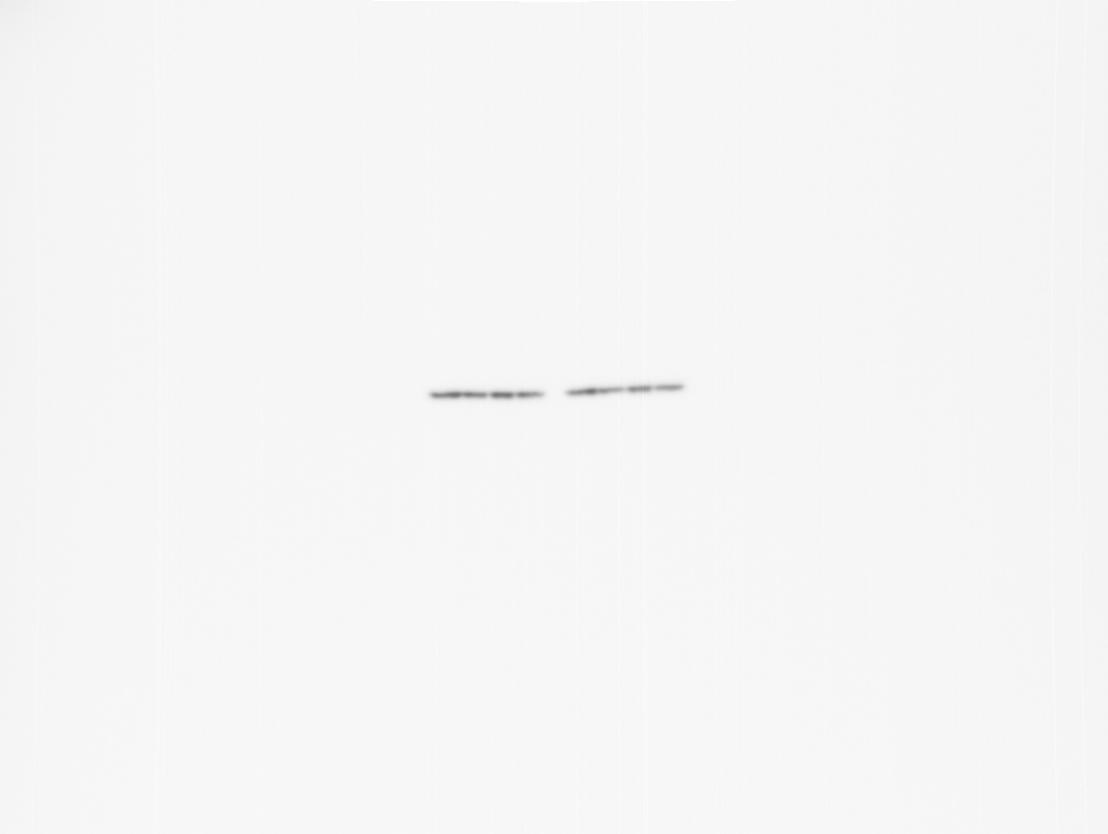

Supplement: Figure 1—figure supplement 1—source data 1. [file elife-81923-fig1-figsupp1-data1.zip › Figure 1-figure supplement 1 -source data/Figure 1-figure supplement 1C GAPDH.jpg]

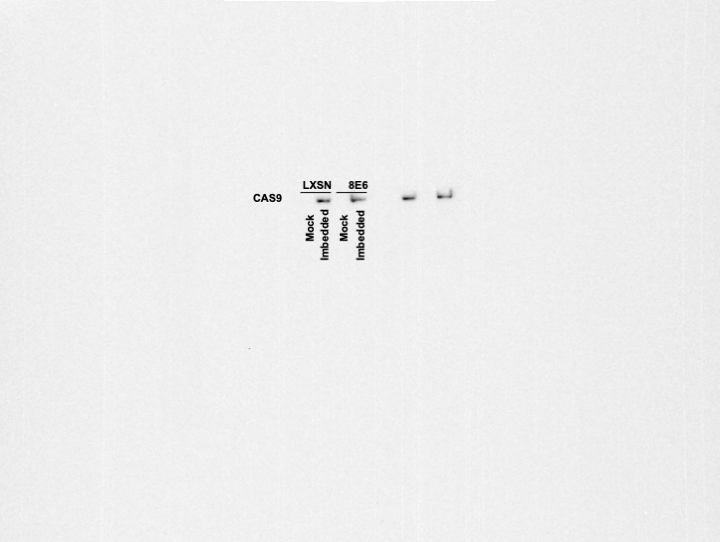

Supplement: Figure 1—figure supplement 1—source data 1. [file elife-81923-fig1-figsupp1-data1.zip › Figure 1-figure supplement 1 -source data/Figure 1-figure supplement 1C CAS9.tiff]

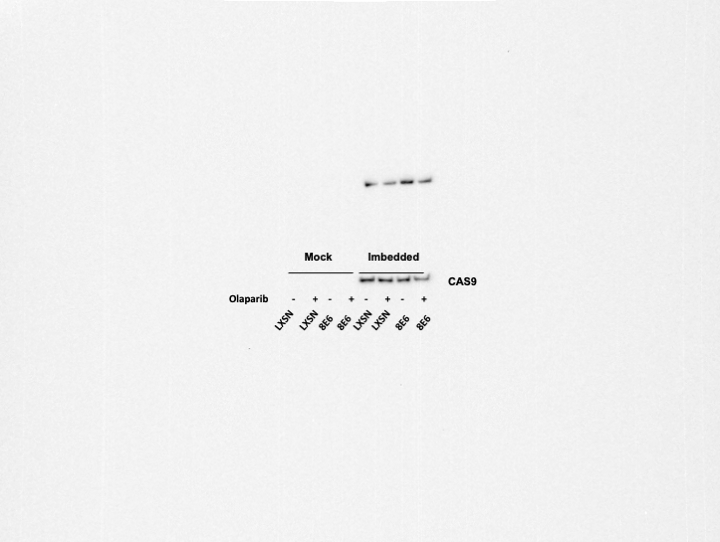

Supplement: Figure 2—figure supplement 1—source data 1. [file elife-81923-fig2-figsupp1-data1.zip › Figure 2-figure supplement 1/Figure 2-figure supplement 1C CAS9.tiff]

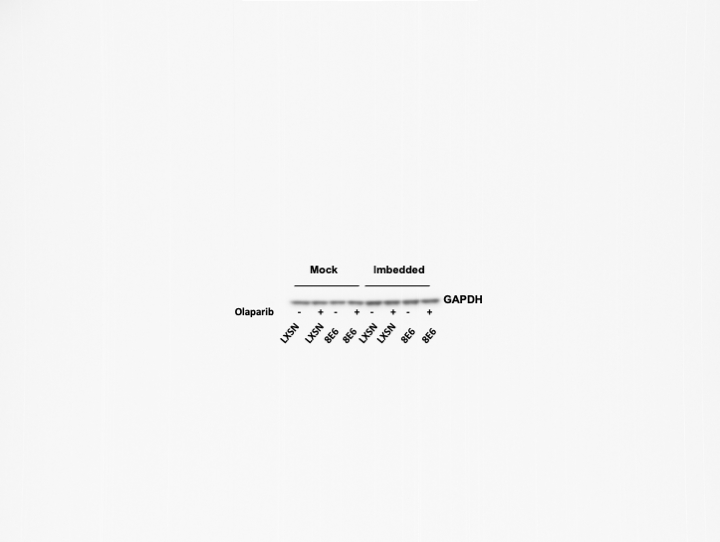

Supplement: Figure 2—figure supplement 1—source data 1. [file elife-81923-fig2-figsupp1-data1.zip › Figure 2-figure supplement 1/Figure 2-figure supplement 1C GAPDH.tiff]

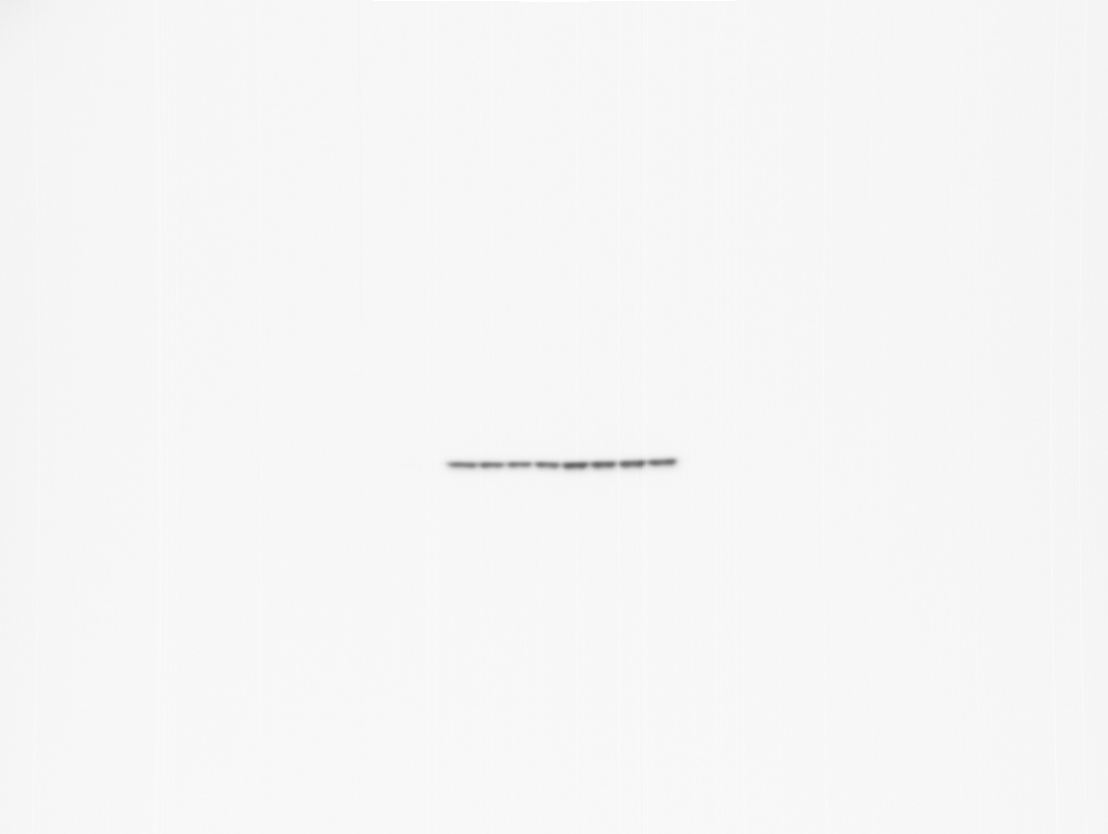

Supplement: Figure 2—figure supplement 1—source data 1. [file elife-81923-fig2-figsupp1-data1.zip › Figure 2-figure supplement 1/Figure 2-figure supplement 1C GAPDH.jpg]

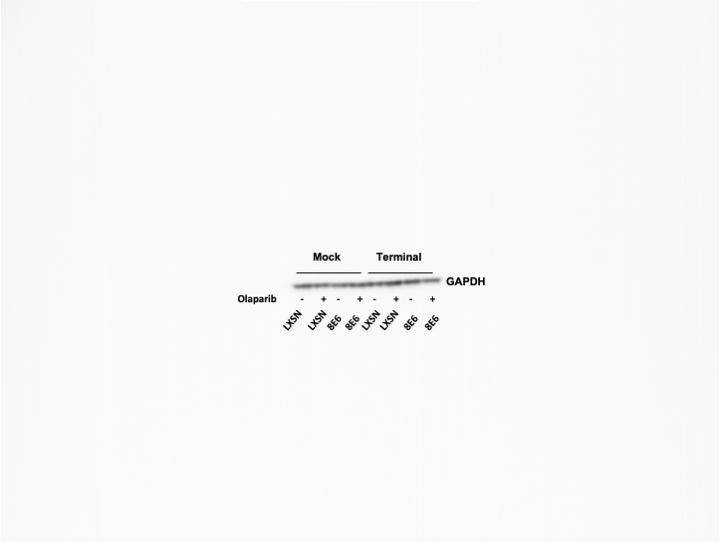

Supplement: Figure 2—figure supplement 1—source data 1. [file elife-81923-fig2-figsupp1-data1.zip › Figure 2-figure supplement 1/Figure 2-figure supplement 1A GAPDH.tiff]

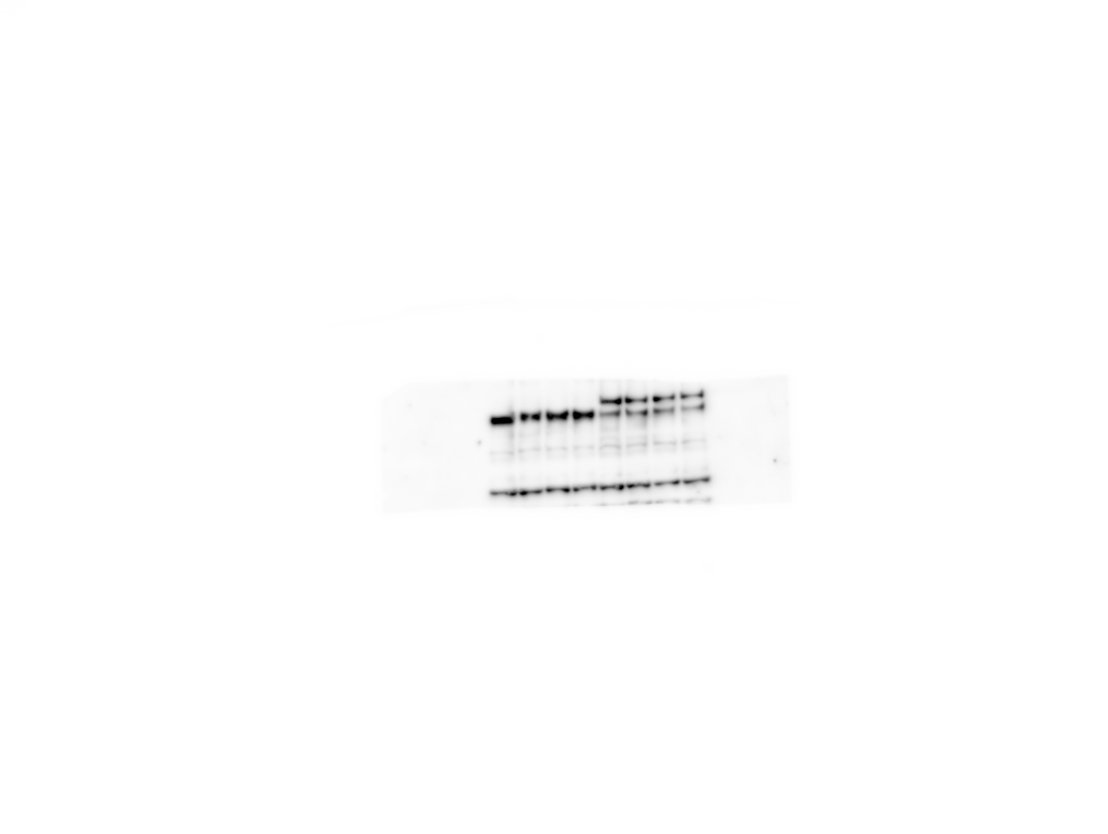

Supplement: Figure 2—figure supplement 1—source data 1. [file elife-81923-fig2-figsupp1-data1.zip › Figure 2-figure supplement 1/Figure 2-figure supplement 1A CAS9.jpg]

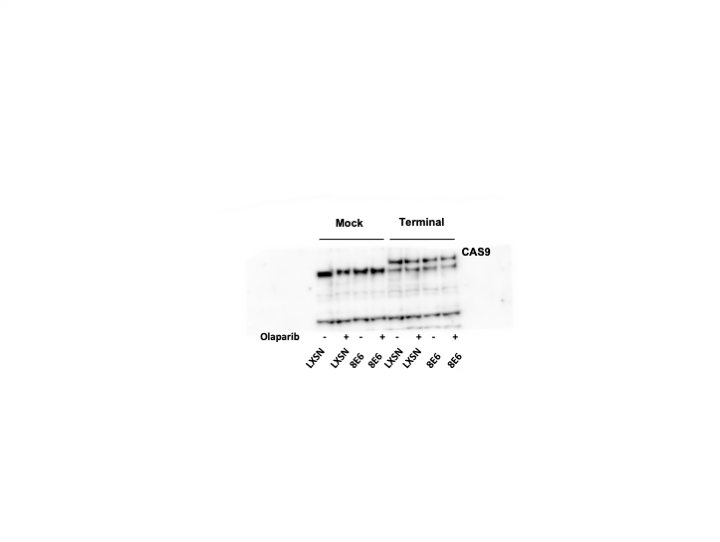

Supplement: Figure 2—figure supplement 1—source data 1. [file elife-81923-fig2-figsupp1-data1.zip › Figure 2-figure supplement 1/Figure 2-figure supplement 1A CAS9.tiff]

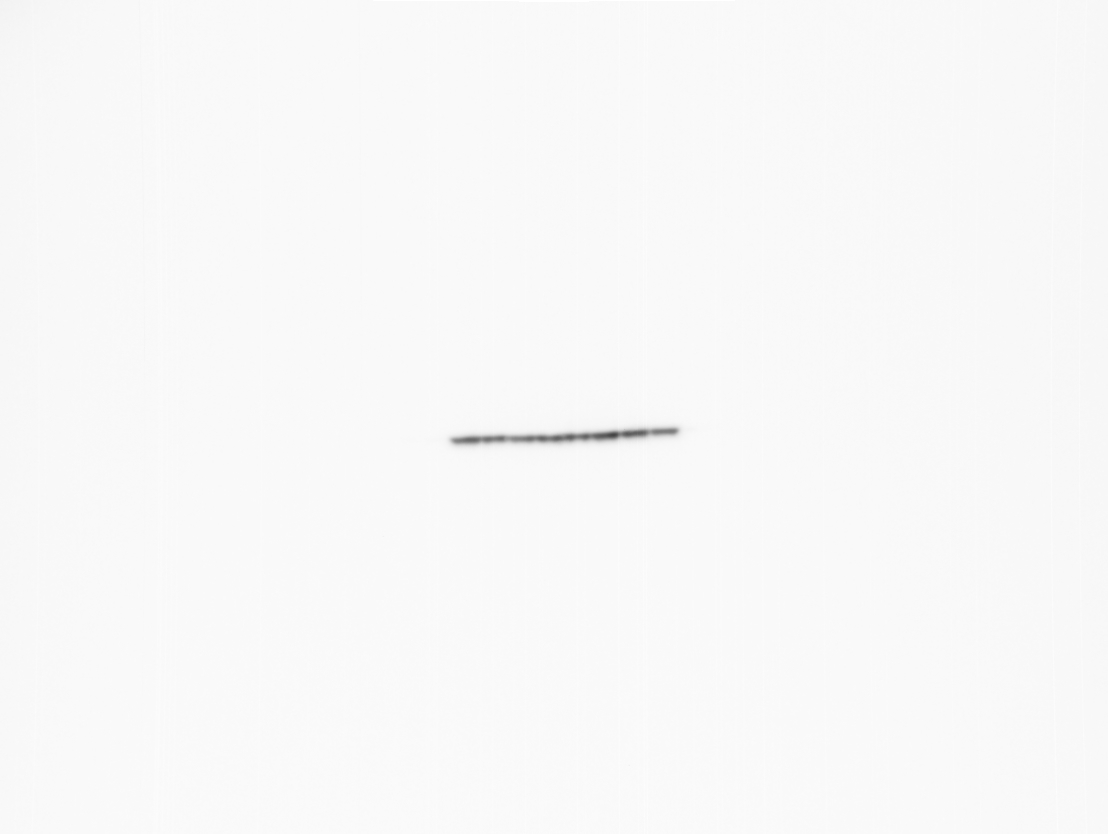

Supplement: Figure 2—figure supplement 1—source data 1. [file elife-81923-fig2-figsupp1-data1.zip › Figure 2-figure supplement 1/Figure 2-figure supplement 1A GAPDH.jpg]

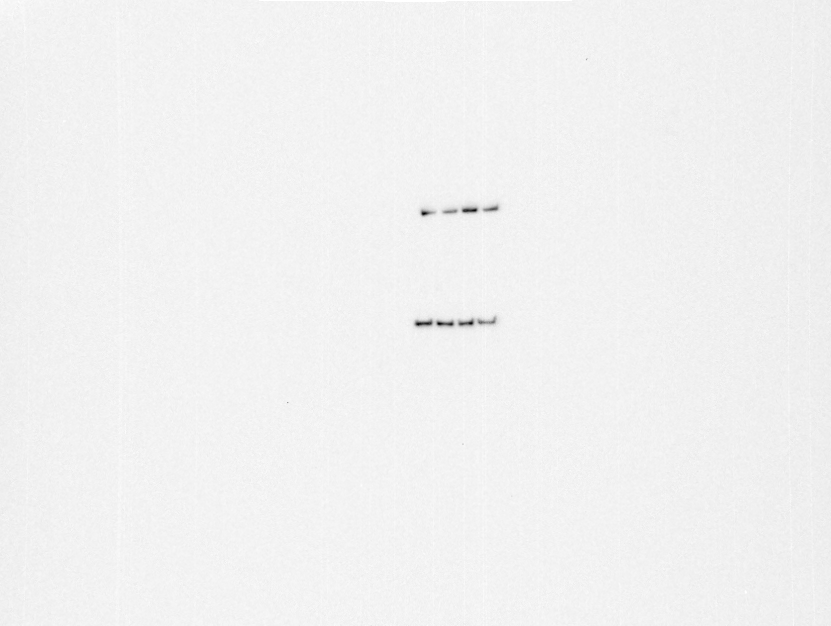

Supplement: Figure 2—figure supplement 1—source data 1. [file elife-81923-fig2-figsupp1-data1.zip › Figure 2-figure supplement 1/Figure 2-figure supplement 1C CAS9.jpg]

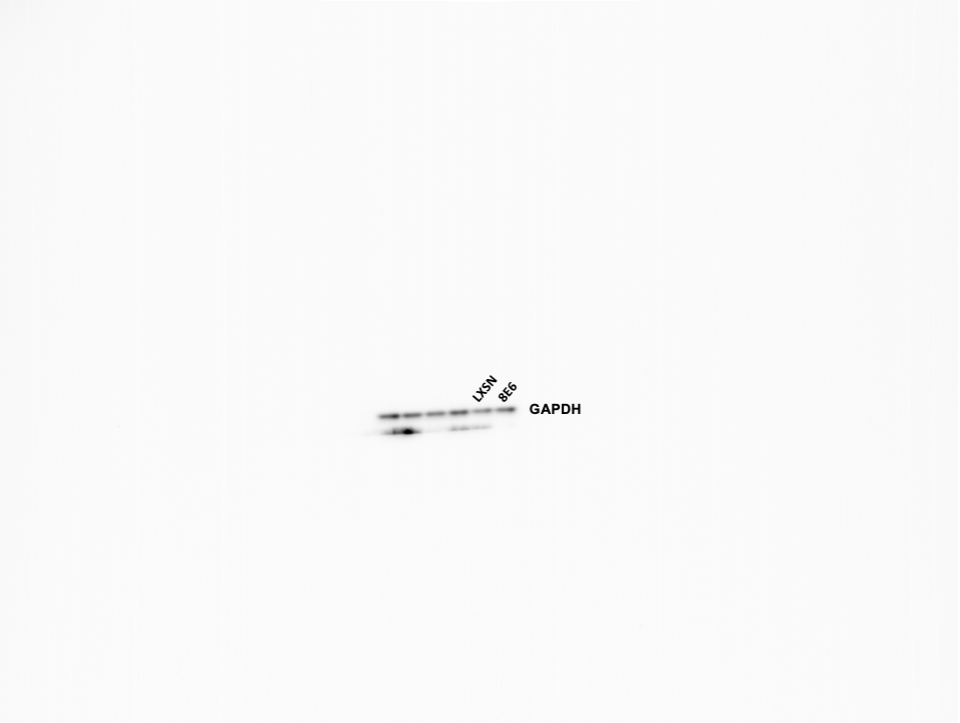

Supplement: Figure 3—source data 1. [file elife-81923-fig3-data1.zip › Figure 3-source data/Figure 3A GAPDH.tiff]

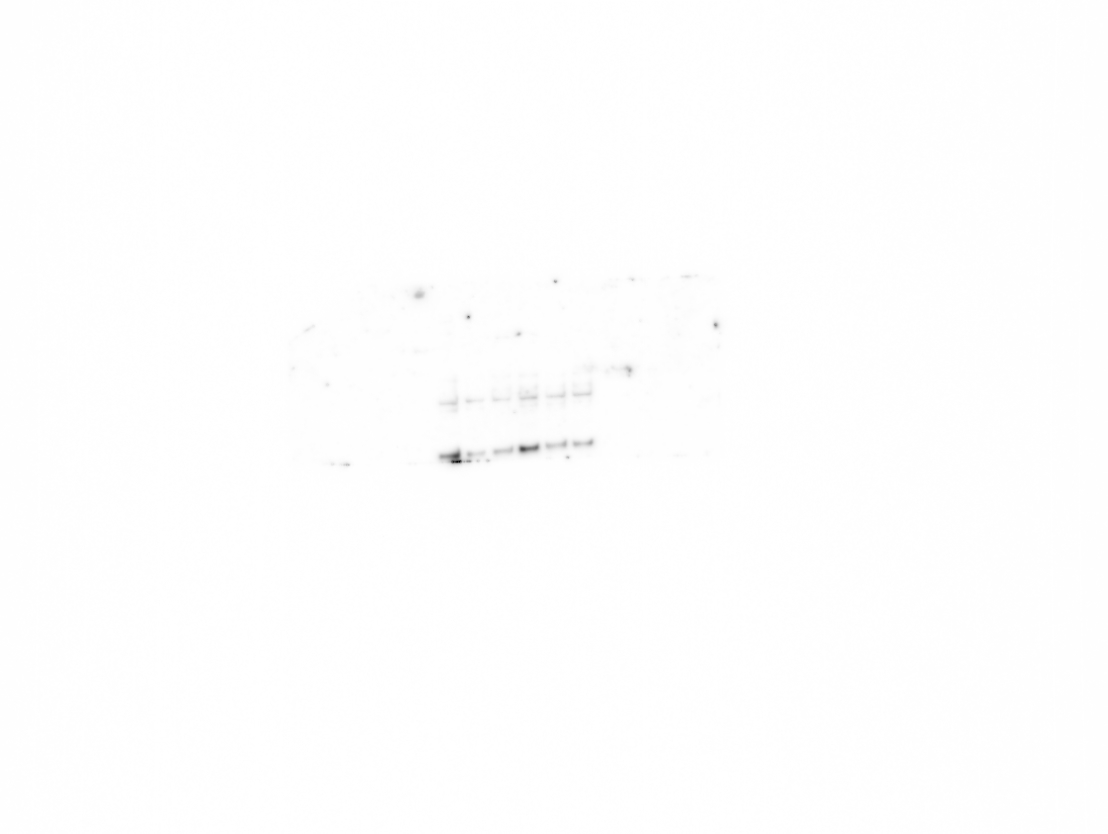

Supplement: Figure 3—source data 1. [file elife-81923-fig3-data1.zip › Figure 3-source data/Figure 3A Pol theta.jpg]

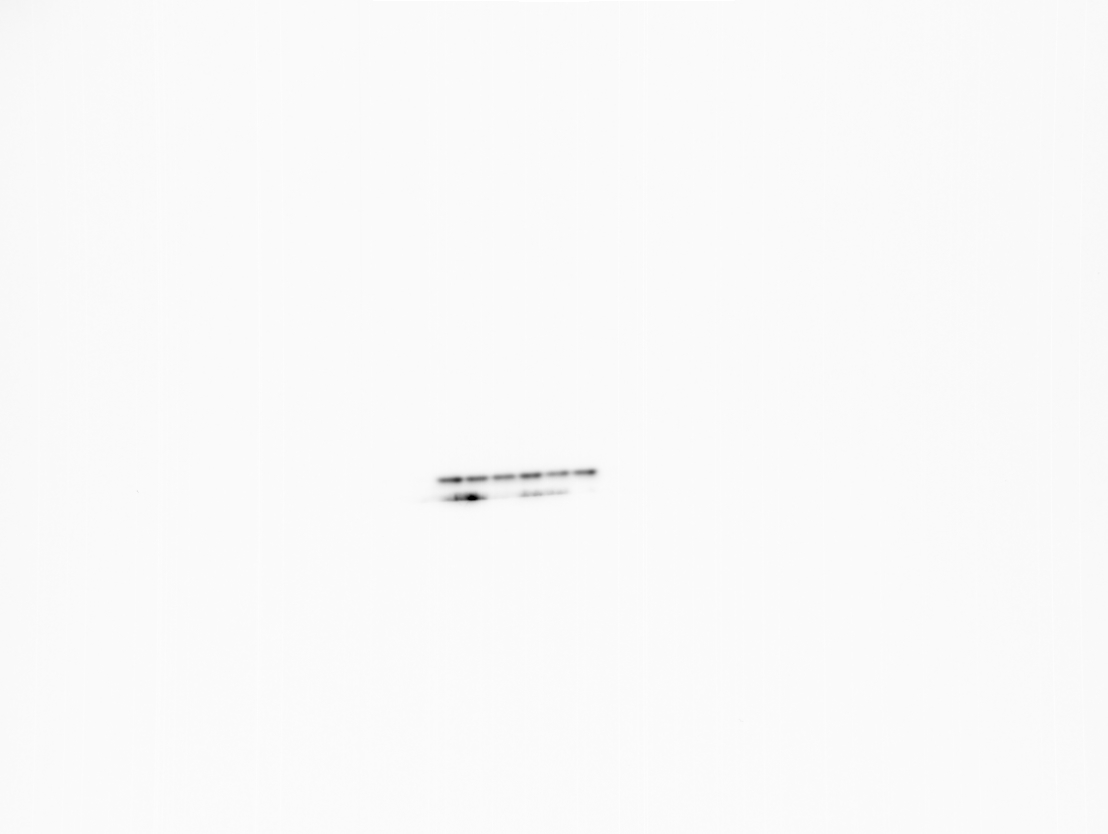

Supplement: Figure 3—source data 1. [file elife-81923-fig3-data1.zip › Figure 3-source data/Figure 3A GAPDH.jpg]

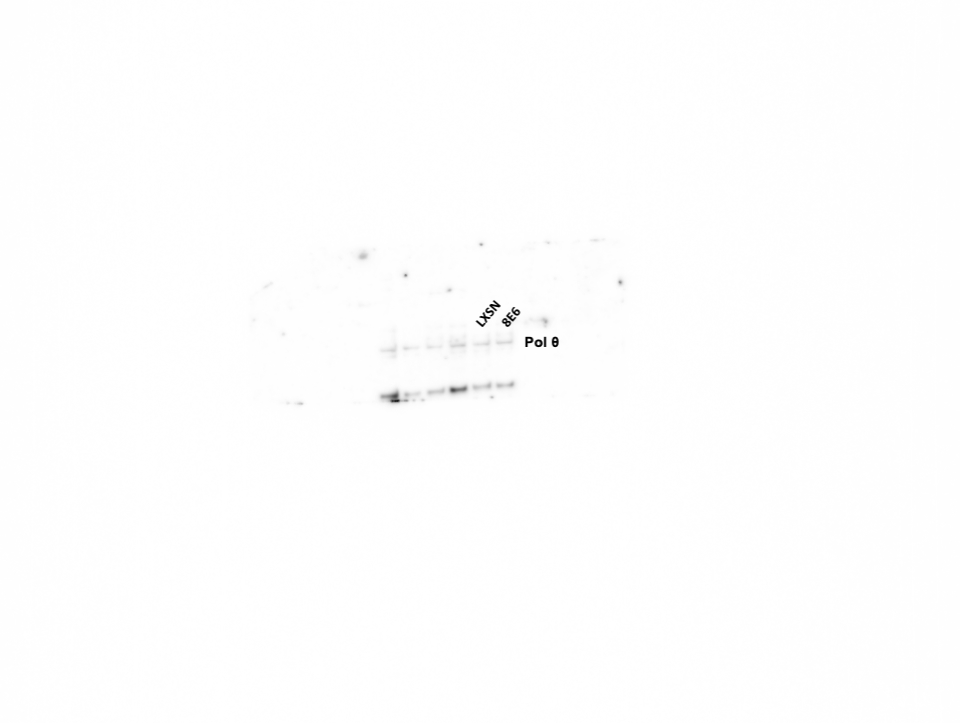

Supplement: Figure 3—source data 1. [file elife-81923-fig3-data1.zip › Figure 3-source data/Figure 3A Pol theta.tiff]

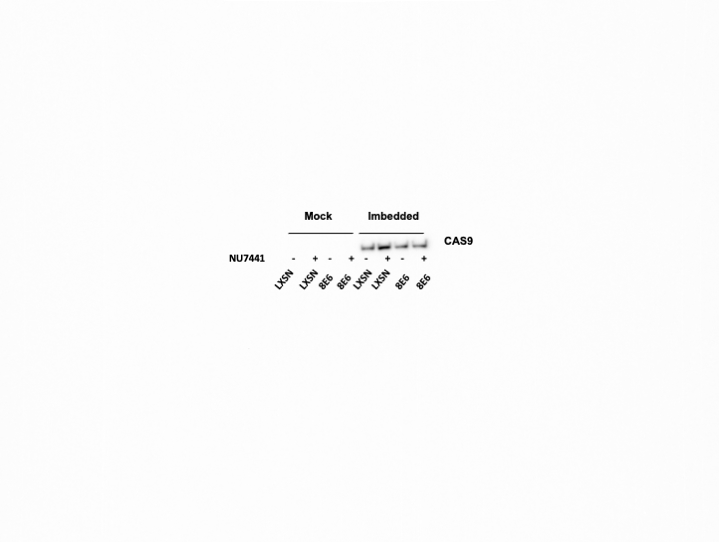

Supplement: Figure 4—figure supplement 1—source data 1. [file elife-81923-fig4-figsupp1-data1.zip › Figure 4-figure supplement 1-source data/Figure 4-figure supplement 1C CAS9.tiff]

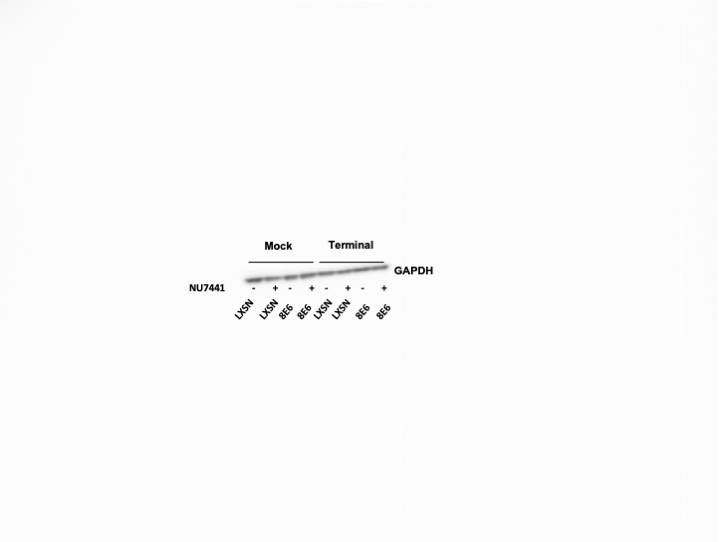

Supplement: Figure 4—figure supplement 1—source data 1. [file elife-81923-fig4-figsupp1-data1.zip › Figure 4-figure supplement 1-source data/Figure 4-figure supplement 1A GAPDH.tiff]

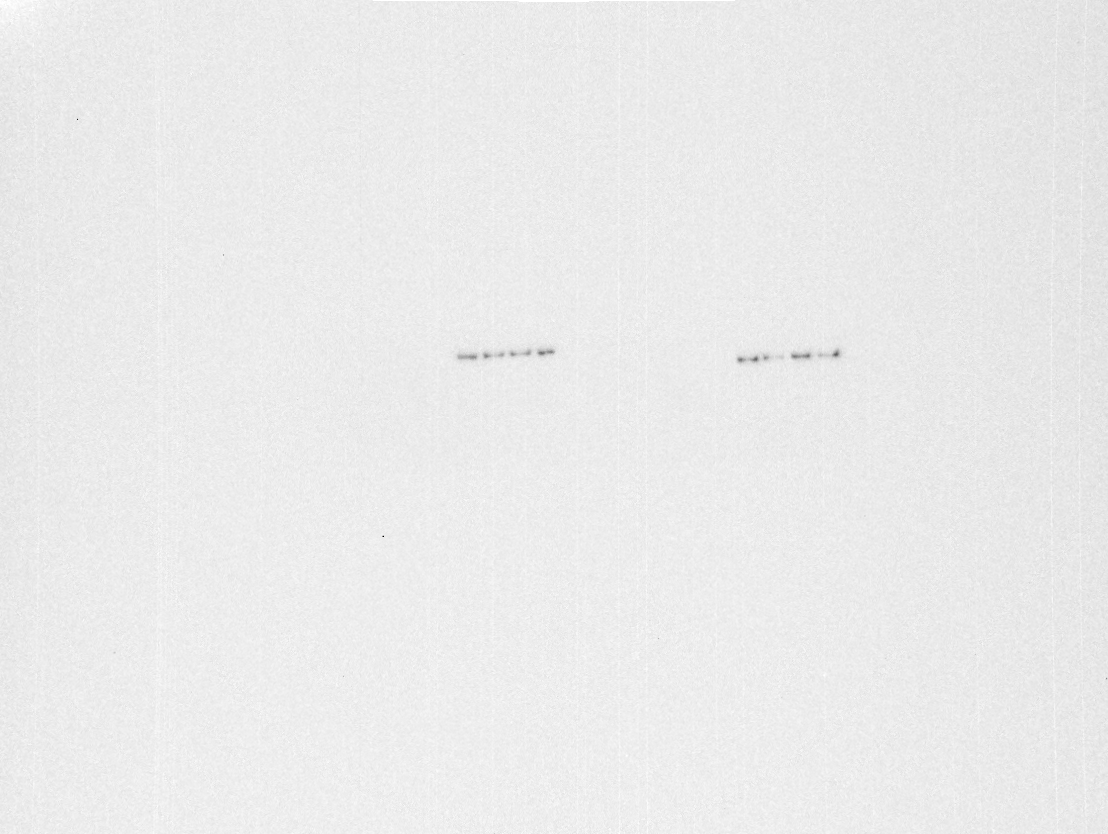

Supplement: Figure 4—figure supplement 1—source data 1. [file elife-81923-fig4-figsupp1-data1.zip › Figure 4-figure supplement 1-source data/Figure 4-figure supplement 1A CAS9.jpg]

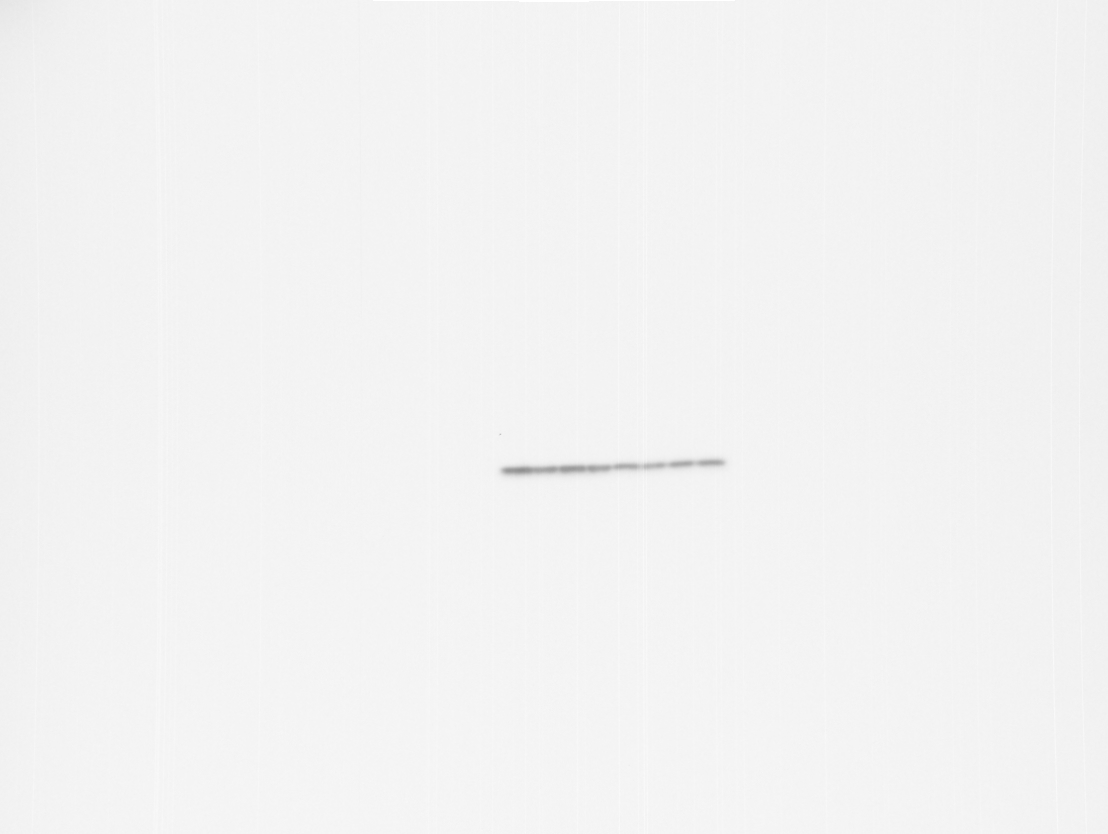

Supplement: Figure 4—figure supplement 1—source data 1. [file elife-81923-fig4-figsupp1-data1.zip › Figure 4-figure supplement 1-source data/Figure 4-figure supplement 1C GAPDH.jpg]

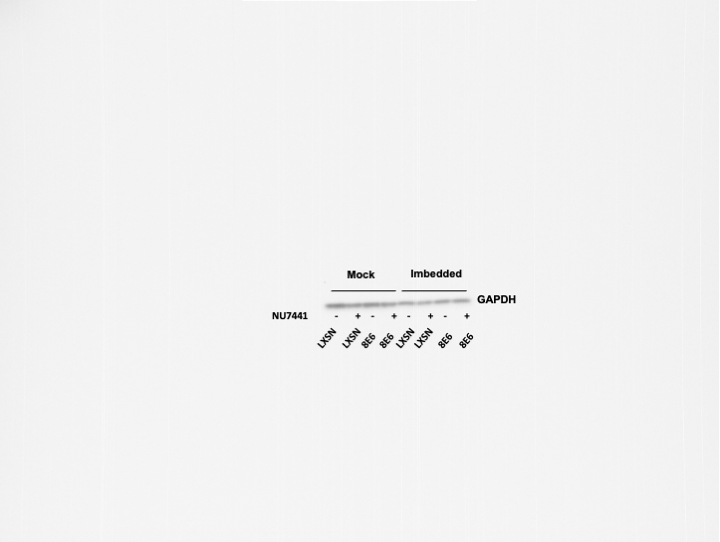

Supplement: Figure 4—figure supplement 1—source data 1. [file elife-81923-fig4-figsupp1-data1.zip › Figure 4-figure supplement 1-source data/Figure 4-figure supplement 1C GAPDH.tiff]

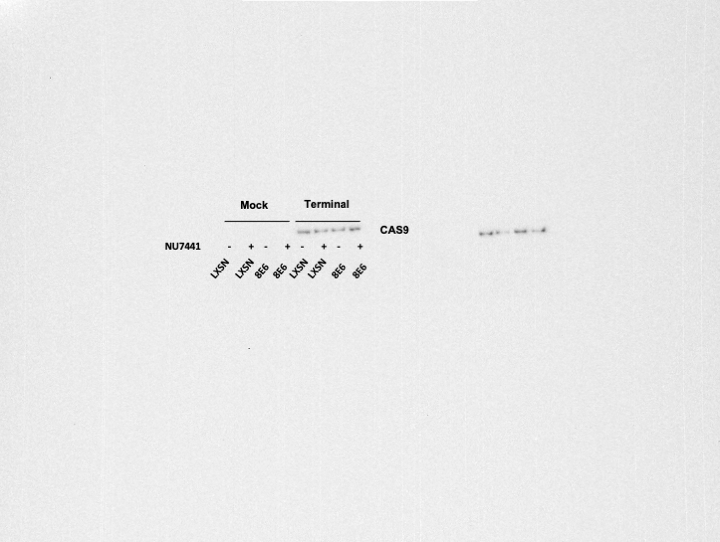

Supplement: Figure 4—figure supplement 1—source data 1. [file elife-81923-fig4-figsupp1-data1.zip › Figure 4-figure supplement 1-source data/Figure 4-figure supplement 1A CAS9.tiff]

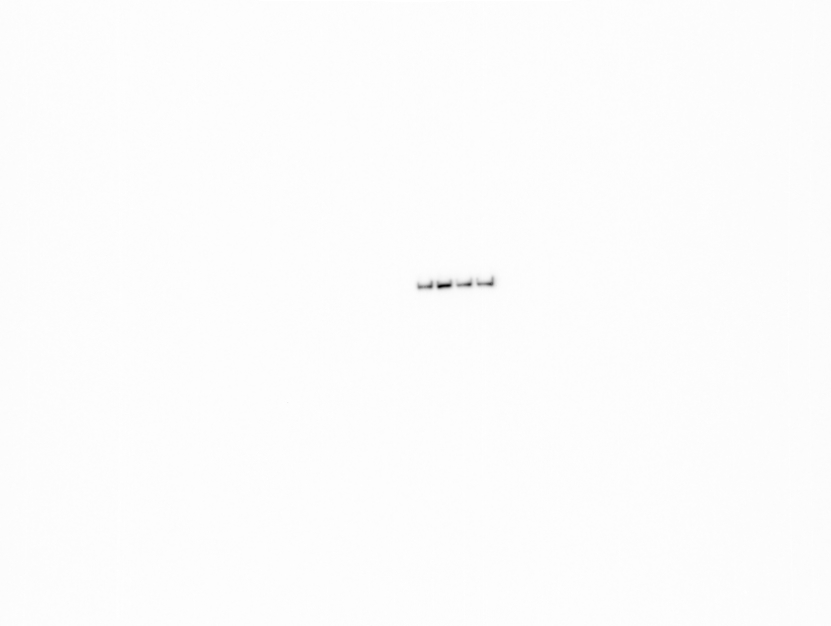

Supplement: Figure 4—figure supplement 1—source data 1. [file elife-81923-fig4-figsupp1-data1.zip › Figure 4-figure supplement 1-source data/Figure 4-figure supplement 1C CAS9.jpg]

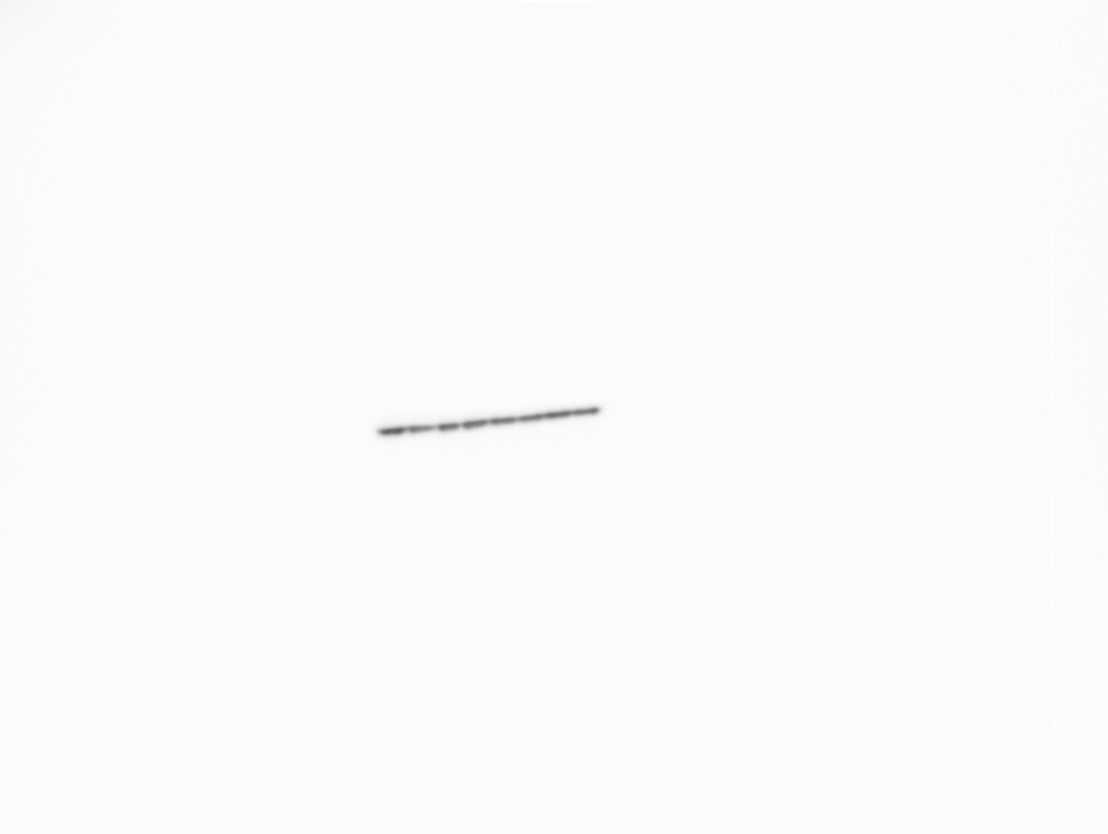

Supplement: Figure 4—figure supplement 1—source data 1. [file elife-81923-fig4-figsupp1-data1.zip › Figure 4-figure supplement 1-source data/Figure 4-figure supplement 1A GAPDH.jpg]
